# Central systolic blood pressure and central pulse pressure predict left ventricular hypertrophy in hypertensive children

**DOI:** 10.1007/s00467-018-4136-7

**Published:** 2018-11-13

**Authors:** Mieczysław Litwin, Łukasz Obrycki, Anna Niemirska, Jędrzej Sarnecki, Zbigniew Kułaga

**Affiliations:** 1 Department of Nephrology and Arterial Hypertension, The Children’s Memorial Health Institute, Aleja Dzieci Polskich 20, 04-730 Warsaw, Poland; 2 Department of Radiology, The Children’s Memorial Health Institute, Warsaw, Poland; 3 Department of Public Health, The Children’s Memorial Health Institute, Warsaw, Poland

**Keywords:** Primary hypertension, Children, Central blood pressure, Left ventricular hypertrophy, Augmentation pressure, Isolated systolic hypertension

## Abstract

**Background:**

Central systolic and pulse pressures are stronger predictors of cardiovascular risk and hypertensive organ damage than brachial blood pressure. It is suggested that isolated systolic hypertension typically seen in adolescents is associated with normal central blood pressure and does not lead to organ damage and this phenomenon is called spurious hypertension.

**Methods:**

We assessed the prevalence of spurious hypertension and analyzed utility of pulse wave analysis as determinant of hypertensive organ damage in 294 children (62 girls; 15.0 ± 2.4 years) diagnosed as primary hypertension. White coat hypertension, ambulatory prehypertension, ambulatory hypertension, and severe ambulatory hypertension were diagnosed in 127, 29, 41, and 97 patients, respectively.

**Results:**

Normal central blood pressure was found in 100% in patients with white coat hypertension, 93% in pre-hypertensives, 51.2% in those with ambulatory hypertension, and 27.8% with severe ambulatory hypertension (*p* = 0.0001). Children with severe ambulatory hypertension had higher central systolic and pulse pressure, pulse wave velocity, and greater prevalence of left ventricular hypertrophy than white coat and prehypertensive children (*p* < 0.05). Left ventricular mass index and carotid intima-media thickness correlated with central systolic and pulse pressure (*p* < 0.05 for all). Receiver operating curve area was similar for augmentation pressure (0.5836), 24-h ambulatory systolic blood pressure (0.5841), central systolic blood pressure (0.6090), and central pulse pressure (0.5611) as predictors of left ventricular hypertrophy.

**Conclusions:**

These findings suggest that pulse wave analysis is complementary to ambulatory blood pressure monitoring in assessment of risk of organ damage in hypertensive adolescents.

## Introduction

By definition, arterial hypertension is based on brachial artery blood pressure measurements. Because of the white coat effect, elevated blood pressure found by means of office or home measurements should be confirmed by 24-h ambulatory blood pressure monitoring (ABPM) [1, 2]. Studies both in adults and in children with primary hypertension (PH) have shown that systolic blood pressure (SBP) and pulse pressure (PP) are risk factors of hypertensive target organ damage (TOD) expressed as left ventricular hypertrophy (LVH) and increased carotid intima-media thickness (cIMT) [3–6]. However, brachial blood pressure is only a surrogate marker of central, i.e., aortic blood pressure. Studies in hypertensive adults have shown that cardiovascular mortality and hypertensive TOD better correlate with central systolic blood pressure (cSBP) and central pulse pressure (cPP) than brachial artery blood pressure [7–10]. cSBP and cPP may be assessed noninvasively by a pulse wave analysis (PWA) from brachial artery. Besides cSBP, there are some other parameters of PWA such as augmentation pressure (AugPress) and augmentation index (AugInd) which reflect the impact of backward pulse pressure from the periphery to aorta. AugPress is cPP ascribed to reflection wave from the peripheral arteries. AugInd is the relative contribution of backward waves on the cPP [11]. It was found that some young subjects diagnosed with isolated systolic hypertension (ISH) based on brachial SBP have normal cSBP and no TOD [12]. This phenomenon has been called spurious hypertension and is believed to be caused by greater amplitude of more elastic brachial arteries in young healthy people in reaction to backward wave [13]. Thus, it was suggested that assessment of cSBP may have greater importance in young people than in adults [14]. Although phenomenon of spurious hypertension is believed to be typical for young people, this phenomenon was not studied in hypertensive children, and ABPM is still the reference method for confirming the diagnosis of arterial hypertension. Moreover, studies analyzing the phenomenon of spurious hypertension included mainly young adults and only small numbers of adolescents despite the fact that the dominant pattern of PH in adolescents is ISH. According to recently published pediatric guidelines, measurement and validation of the usefulness of PWA, including cSBP and cPP, in the diagnosis of arterial hypertension and assessment of cardiovascular risk is still a challenge for future studies [1, 2]. Thus, the aims of our study were to assess prevalence of spurious hypertension among children diagnosed as PH, to analyze the usefulness of PWA as determinant of hypertensive TOD and to compare cSBP, cPP, AugPress, and AugInd with ABPM as determinants of hypertensive TOD in children with PH.

## Patients and methods

Two-hundred and ninety-four children (62 girls; 15.0 ± 2.4 years) referred with the diagnosis of arterial hypertension based on office blood pressure readings exceeding 95th percentile for age, sex, and height on at least three independent occasions and confirmed by auscultatory blood pressure measurements were included in the study. None of the patients received antihypertensive drugs. All patients underwent full diagnostic evaluation according to the recently published pediatric guidelines of the European Society of Hypertension [2] including assessment of 24-h ABPM, left ventricular mass index (LVMi), cIMT, pulse wave velocity (PWV), and PWA. PWV and PWA were assessed by means of the oscillometric device (Vicorder®). Office blood pressure was measured with validated oscillometric device (Datascope Accutor Plus, Datascope Corp., Fairfield, NJ, USA) according to recently published guidelines [2] on a day of ABPM and at time of TOD evaluation. Exclusion criteria were a diagnosis of secondary hypertension and/or anatomical abnormalities of arterial tree (e.g., coarctation of aorta, mid-aortic syndrome or stenosis of subclavian arteries), acute infection in preceding 6 weeks, and any chronic condition other than PH.

### ABPM measurements

All ABPM measurements were assessed oscillometrically by means of the SpaceLabs Monitor 90207 using the most appropriate cuff fitted to the non-dominant arm. Readings were taken every 20 min during daytime and every 30 min at night. Recordings lasting ≥ 20 h with ≥ 80% of readings were considered valid and were included in the analysis. Patients completed a diary for the identification of activity (wake) and sleep periods.

We used a recently published classification system based on ABPM to classify patients as having white coat hypertension (WCH), ambulatory prehypertension (AmbpreHT), ambulatory hypertension (AmbHT), and severe ambulatory hypertension (AmbsevereHT) [15]. In short, WCH was defined as office blood pressure ≥ 95 percentile, mean ABPM SBP and diastolic blood pressure (DBP) below 95th percentile, and SBP/DBP load below 25%. AmbpreHT was defined as office BP ≥ 120/80 (or ≥ 90th percentile—in fact, all patients had office blood pressure > 95th percentile), ABPM < 95th percentile and SBP/DBP load ≥ 25%. AmbHT was defined as office blood pressure ≥ 95th percentile (or ≥ 140/90 mmHg for adolescents 16 years old and older) and SBP/DBP load 25–50%, and SevereambHT as office blood pressure ≥ 95th percentile (or ≥ 140/90 mmHg for adolescents 16 years old and older) and SBP/DBP load > 50%.

### Measurement of carotid to femoral PWV and PWA

**Fig. 1:**
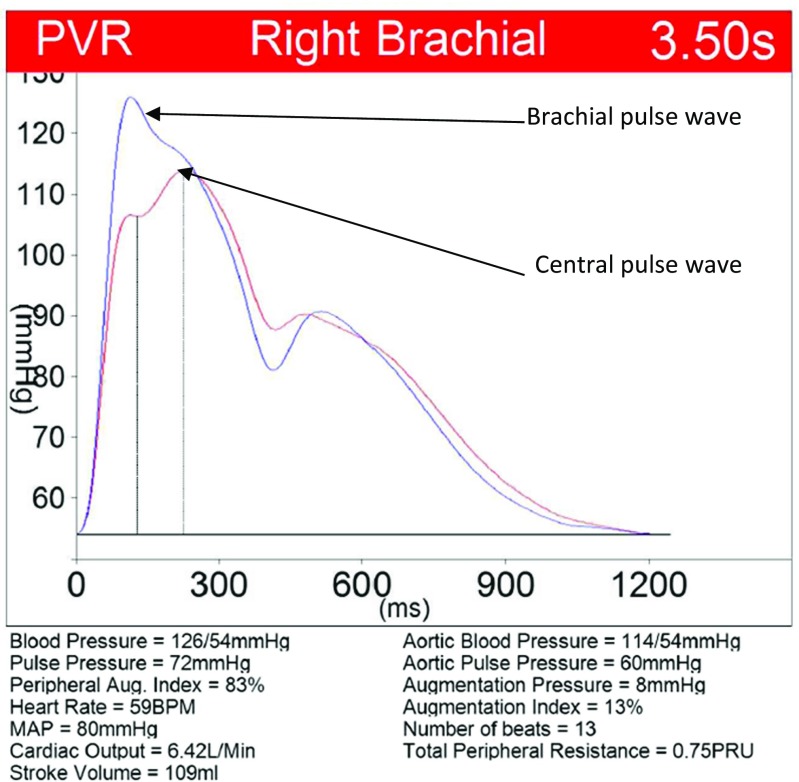
Graphic presentation of pulse wave analysis obtained by Vicorder®. *MAP* mean arterial pressure

Pulse wave velocity and PWA were measured non-invasively with oscillometric method using Vicorder® (SMT Medical) system device. This system has been validated against applanation tonometry systems (Sphygmocor®) and invasive measurements of central blood pressure. It was found to be a reliable and simple alternative to tonometry [16, 17]. Moreover, cSBP by Vicorder was more closely related to invasive measurements than with tonometry measurements. In addition, this method is investigator-independent and is recommended in studies of large groups of subjects [18]. Vicorder® has been also validated in pediatric studies [18]. Graphic presentation of the main parameters used in PWA is shown in Fig. 1.

The Vicorder system provides a simple and quick non-invasive oscillometric method of obtaining PWV for an arterial segment. Measurement was performed in the supine position after 5 min of rest by means of the Vicorder device according to the actual guidelines [19, 20]. A 100-mm wide cuff was placed around the right upper thigh to measure the femoral pulse wave and a 30-mm plethysmographic partial inflatable sensor was placed over the carotid region, able to pick up the carotid pulse wave. Both cuffs are automatically inflated to 65 mmHg, and the corresponding oscillometric signal from each cuff is digitally analyzed by means of the latest patented technique to accurately extract, in real time, the pulse time delay and the consequent PWV.

The waveform of brachial artery pulse obtained oscillometrically was analyzed and then by means of the transfer function the aortic waveform was calculated. The analysis of a waveform of the aortic wave enables calculation of some parameters describing the characteristics of the arterial system including aortic central blood pressure, AugPress, AugInd, cPP, cardiac output, and total peripheral resistance [19–21]. Both for PWV and PWA, first few waves were omitted and when next at least 5 pulse waves were of good quality 10–15 consecutive pulse waves (heart beats) were taken to analysis (Fig. 1).

### Echocardiography

All echocardiography examinations were performed by 1 examiner who knew the clinical diagnosis, but was not aware of the severity of hypertension and the results of PWA. Echocardiography measurements were performed according to the American Society of Echocardiography guidelines [22, 23]. To standardize the left ventricular mass to height, left ventricular mass index (LVMi) was calculated according to de Simone formula [24]. LVH was defined as an LVMi value above the 95th percentile for age- and sex-based reference data [23].

### cIMT and wall cross-sectional area (WCSA) of carotid arteries’ measurements

Carotid intima-media thickness was evaluated by ultrasound according to the methodology described previously. The mean WCSA was calculated from the equation: WCSA = π (dD/2 + IMT) 2 − π (dD/2) 2, where dD is the mean diastolic diameter [25, 26].

### Laboratory investigations

Plasma glucose level, lipid profile, and serum uric acid were assessed at diagnosis. Blood samples were taken after 12 h of fasting. After separation of serum without anticoagulant at vacuum tubes, routine clinical chemistry parameters were measured with use of Cobas 6000® (Roche, Switzerland). We used unit c501 as biochemistry analyzer for spectrophotometric, immunoturbidimetric, and ion-selective determination of biochemical analytes: uric acid, ions, C-reactive protein, glucose, lipids, and creatinine.

### Statistical analysis

The anthropometrical indices, cIMT, WCSA, LVMi, and PWV values, were expressed as absolute values and standard deviation score (SDS) from the mean of the normal values according to the referential normative values published recently [24–27]. The homogeneity of variance was checked with the Shapiro–Wilk test. Continuous variables with a normal distribution were compared by means of the Student test for independent variables. Continuous values with non-parametric distribution were compared by means of the Wilcoxon test. Multigroup comparisons were completed with the ANOVA test with Bonferroni correction.

Variables with normal distribution were presented as mean and SD values, whereas variables with non-parametric distribution were presented as median and range values between the 5th and 95th percentiles. The correlation analysis was performed by means of the Spearman test for non-parametric distribution. Variables with significant correlation including changes in anthropometrical parameters and changes in BP and metabolic parameters were then included in the step-wise multiple regression analysis. *p* values < 0.05 were considered statistically significant, and values between 0.05 and 0.1 were considered as demonstrating trend toward significance.

Sensitivity and specificity of cSBP, cPP, AugPress, AugInd, and ABPM as predictors of TOD were determined by means of the receiver operator curve (ROC) analysis.

## Results

**Fig. 2:**
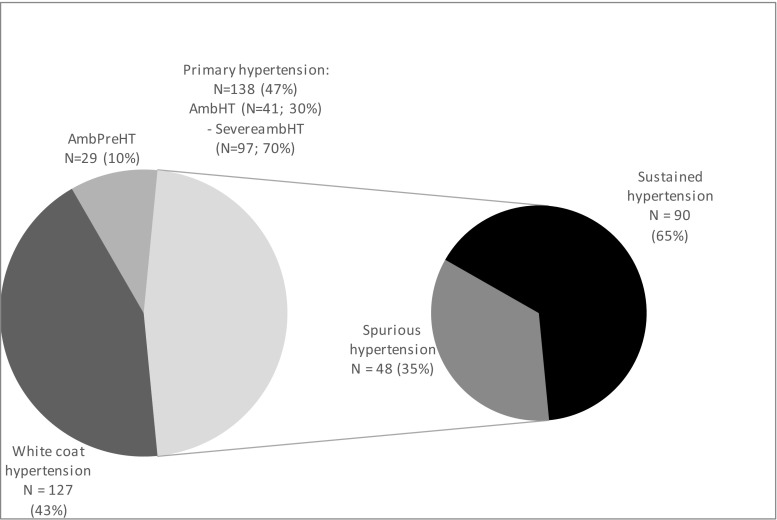
Blood pressure status based on ambulatory blood pressure monitoring (ABPM) and central systolic blood pressure (cSBP) measurements. *AmbpreHT* ambulatory prehypertension, *AmbHT* ambulatory hypertension, *SevereambHT* severe ambulatory hypertension

Out of 308 children referred because of the diagnosis of arterial hypertension based on at least three independent office blood pressure measurements and in whom secondary causes of arterial hypertension were excluded, 294 patients in whom all measurements have been completed were included in the analysis. In 127 (43.2%) subjects, WCH was diagnosed and 29 had AmbpreHT (9.8%). In 138 (47%) patients, the diagnosis of arterial hypertension was confirmed by ABPM, and ultimately, PH was diagnosed. In 41 (29.7%) of them AmbHT and in 97 (70.3%) SevereambHT was diagnosed (Fig. 2). Out of 138 patients diagnosed as PH, 92 (66%) had ISH, 6 (4%) isolated diastolic hypertension (IDH), and 40 (29%) had systo-diastolic hypertension (SDH). The groups did not differ regarding age, sex distribution, anthropometrical parameters (Table 1), and biochemical values (data not shown). Hypertensive children had significantly greater cSBP, cPP, cIMT, and greater prevalence of LVH than children with WCH and AmbpreHT (Table 1).

**Table 1:** Characteristic of patients group

	Normal ABPM (WCH) *N* = 127	AmpreHT *N* = 29	AmbHT *N* = 41	SevereambHT *N* = 97	*p*
Age	14.8 ± 2.3	14.7 ± 2.7	15.0 ± 3.0	15.3 ± 2.3	n.s.
Sex	♀ 26 (20.5%)	♀ 5 (17.2%)	♀ 6 (14.6%)	♀ 25 (26%)	n.s.
BMI	24.6 ± 4.7	23.4 ± 4.1	24.8 ± 5.3	24.6 ± 3.6	n.s.
BMI-SDS	1.1 ± 1.0	0.8 ± 1.0	0.9 ± 1.1	1.1 ± 0.8	n.s.
WC (cm)	82.0 ± 11.0	78.0 ± 12.0	82.4 ± 15.2	79.8 ± 8.0	n.s.
WC-SDS	1.2 ± 0.9	0.9 ± 0.8	1.1 ± 1.1	0.9 ± 0.8	n.s.
24 h SBP (mmHg)	121 ± 6	126 ± 5	130 ± 5	137 ± 6	*p* < 0.05 for all comparisons
24 h DBP (mmHg)	68 ± 4	72 ± 5	74 ± 5	76 ± 6	normal ABPM (WCH) vs SevereambHT *p* < 0.03
Pulse wave velocity (m/s)	5.7 ± 0.6	6.0 ± 0.8	5.7 ± 0.7	6.0 ± 0.8	Normal ABPM (WCH) vs SevereambHT; *p* = 0.02
Pulse wave velocity-SDS	1.6 ± 1.2	2.1 ± 1.3	1.7 ± 1.1	2.2 ± 1.6	n.s.
Pulse wave velocity index	0.73 ± 0.13	0.77 ± 0.17	0.73 ± 0.18	0.75 ± 0.16	n.s.
Central systolic blood pressure (mmHg)	114 ± 9	116 ± 10	117 ± 8	122 ± 10	SevereambHT vs normal ABPM (WCH) and AmbpreHT; *p* < 0.0001
Central pulse pressure (mmHg)	47 ± 8	46 ± 6	49 ± 8	51 ± 9	SevereambHT vs normal ABPM (WCH) and AmbpreHT; *p* < 0.004
Augmentation pressure (mmHg)	3.5 ± 2.8	2.8 ± 1.4	3.9 ± 2.6	4.2 ± 2.9	n.s.
Augmentation index	7.0 ± 4.8	6.2 ± 3.0	7.8 ± 5.1	7.9 ± 5.1	n.s.
Carotid intima-media thickness (mm)	0.44 ± 0.03	0.45 ± 0.03	0.44 ± 0.03	0.45 ± 0.04	SevereambHT vs normal ABPM (WCH); *p* = 0.006
Carotid intima-media thickness-SDS	1.05 ± 0.69	1.28 ± 0.70	1.1 ± 0.70	1.38 ± 0.91	SevereambHT vs normal ABPM (WCH); *p* = 0.04
Carotid wall cross sectional area (mm^2^)	7.1 ± 1.0	7.2 ± 0.9	7.2 ± 1.1	7.5 ± 1.2	n.s.
Carotid wall cross sectional area-SDS	0.70 ± 1.0	0.77 ± 0.99	0.73 ± 1.17	1.11 ± 1.43	n.s.
Left ventricular mass index (g/m height^2.7^)	34.8 ± 6.6	34.1 ± 5.1	35.1 ± 5.6	36.6 ± 6.2	n.s.
Left ventricular hypertrophy	27/125 (21.6%)	3/29 (10.3%)	8 /40 (20%)	31/97 (32.0%)	*p* = 0.06 chi-square = 7.124

*AmbpreHT* ambulatory prehypertension, *AmbHT* ambulatory hypertension, *BMI* body mass index, *SDS* standard deviation score, *SevereambHT* severe ambulatory hypertension, *WCH* white coat hypertension, *ABPM* ambulatory blood pressure monitoring

### Pulse wave analysis and central blood pressure

All patients with WCH and 27 of 29 (93.1%) patients with AmbpreHT had normal cSBP in comparison with 21 out of 41 (51.1%) patients with AmbHT and 27 out of 97 (27.8%) patients with SevereambHT (*p* = 0.0001). Overall, 48 of 138 (35%) children in whom ABPM confirmed arterial hypertension had normal cSBP. Out of 92 patients with ISH, 56 (61%) had elevated cSBP, 4 out of 6 (61%) of those with IDH, and 30 out of 40 (75%) patients with SDH.

### Echocardiography and left ventricular hypertrophy

**Fig. 3:**
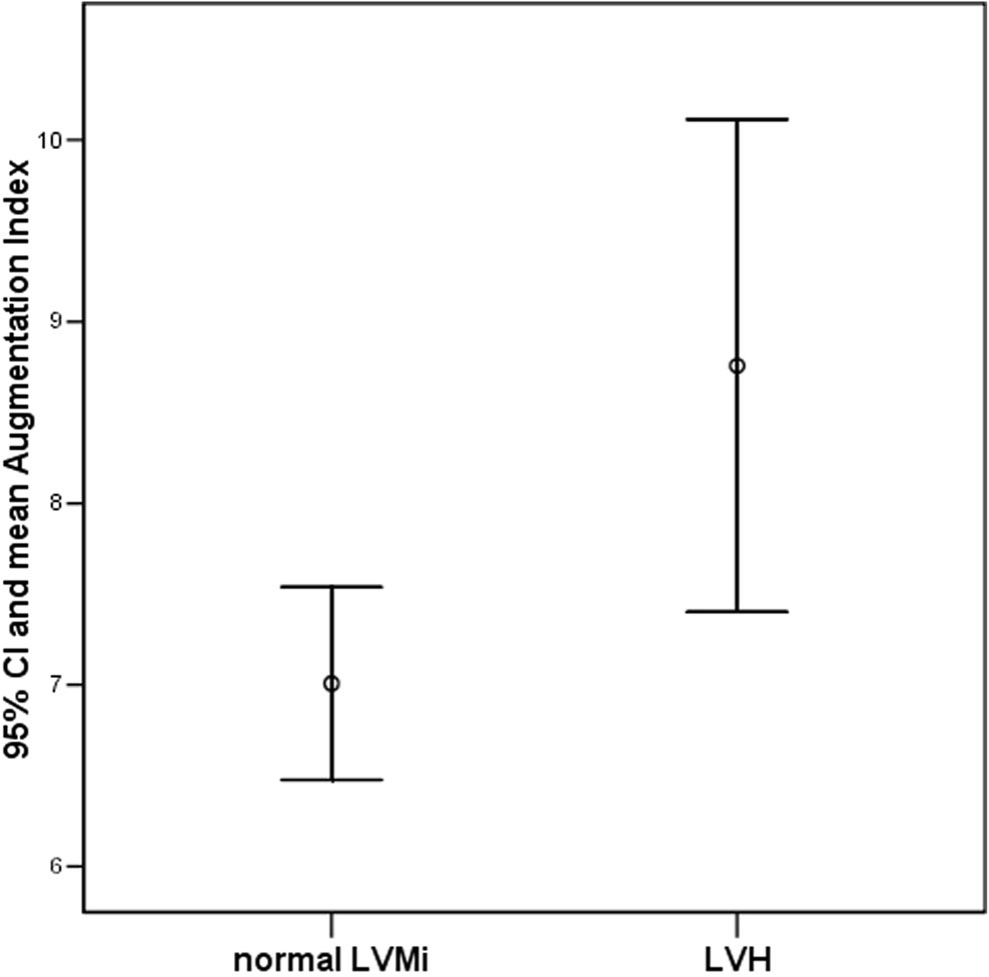
Comparison of augmentation index in children with normal left ventricular mass (*N* = 201) and in children with left ventricular hypertrophy (*N* = 62). (*p* = 0.02). *LVMi* left ventricular mass index, *LVH* left ventricular hypertrophy, *95% CI* 95% confidence interval

In 291 out of 294 patients, results of echocardiographic examination were reliable and LVMi was assessed. Prevalence of LVH increased from 21.6% among WCH patients to 32% in patients with SevereambHT (*p* = 0.06, chi-square = 7.124) (Table 1). Overall, LVH was more prevalent in hypertensive children (AmbHT and SevereambHT) (39 out of 137; 28.4%) than in WCH and prehypertensive children (30 out of 154; 19.4%) (*p* = 0.04, chi-square = 4.036). On the average, patients with LVH had greater BMI-SDS (1.6 ± 0.8 vs 0.8 ± 0.9; *p* = 0.0001), waist-SDS (1.4 ± 0.9 vs 1.0 ± 0.9; *p* = 0.001), 24 h SBP (130 ± 10 vs 127 ± 9 ±; *p* = 0.029), AugPress (4.6 ± 3.7 vs 3.4 ± 2.4 mmHg; *p* = 0.004), AugInd (8.9 ± 6.0 vs 6.9 ± 4.3; *p* = 0.02) (Fig. 3), and lower ratio of peripheral pulse pressure to central pulse pressure (*p* = 0.04). There was a trend to higher cSBP in those with LVH (119 ± 10 vs 116 ± 9 mmHg; *p* = 0.1).

### Associations of central systolic blood pressure and target organ damage

Comparison of all patients with normal (*n* = 202) and elevated cSBP (*n* = 92) revealed significantly greater values of 24 h SBP and DBP, cIMT, LVMi, and PWV-SDS in children with the elevated cSBP (Table 2). When the analysis was restricted only to group of 138 children diagnosed as PH (48 with normal cSBP vs 90 with the elevated cSBP), it was found that cIMT, PWV, and prevalence of LVH were greater in patients with the elevated cSBP in comparison with those who had normal cSBP (Table 2). However, there were no differences regarding 24 h blood pressure values.

**Table 2:** Characteristic of patients with normal and elevated central blood pressure

	Patients with normal central systolic blood pressure *N* = 202	Patients with elevated central systolic blood pressure *N* = 92	*p*	Patients with primary hypertension and normal central SBP *N* = 48	Patients with primary hypertension and elevated central SBP *N* = 90	*p*
Age	15.0 ± 2.4	14.6 ± 2.9	n.s.	15.8 ± 2.2	14.8 ± 2.8	n.s.
Sex	♀40 (19.8%)	♀22 (23.9%)	n.s.	♀7		
(14.6%)	♀22					
(24.4%)	n.s.					
BMI	24.1 ± 4.3	25.1 ± 4.1	n.s.	24.1 ± 3.8	24.9 ± 4.3	n.s.
BMI-SDS	1.0 ± 0.9	1.2 ± 0.9	n.s.	0.7 ± 0.9	1.2 ± 0.8	0.02
WC (cm)	80 ± 11	81 ± 11	n.s.	80 ± 10	81 ± 11	n.s.
Waist circumference-SDS	1.1 ± 0.9	1.1 ± 0.9	n.s.	0.7 ± 1.0	1.1 ± 0.9	0.09
24 h SBP (mmHg)	124 ± 9	135 ± 8	0.0001	134 ± 6	135 ± 8	n.s.
24 h DBP (mmHg)	70 ± 5	76 ± 7	0.0001	74 ± 5	77 ± 7	0.08
24 h heart rate (beat/min.)	75 ± 5	79 ± 12	0.04	76 ± 10	79 ± 12	0.08
Central SBP (mmHg)	114 ± 8	124 ± 9	0.0001	114 ± 7	124 ± 9	0.0001
Central pulse pressure (mmHg)	47 ± 7	52 ± 9	0.0001	48 ± 5	52 ± 10	0.007
Augmentation pressure (mmHg)	3.4 ± 2.5	4.5 ± 3.2	0.002	3.4 ± 2.0	4.5 ± 3.3	0.01
Augmentation index	6.9 ± 4.4	8.6 ± 5.5	0.007	6.8 ± 3.9	8.4 ± 5.4	0.05
Pulse wave velocity (m/s)	5.8 ± 0.7	6.0 ± 0.8	0.01	5.8 ± 0.7	6.0 ± 0.8	0.09
Pulse wave velocity-SDS	1.6 ± 1.2	2.1 ± 1.4	0.009	1.4 ± 1.1	2.3 ± 1.5	0.0001
Carotid intima-media thickness (mm)	0.44 ± 0.03	0.45 ± 0.04	0.003	0.44 ± 0.03	0.45 ± 0.04	0.1
Carotid intima-media thickness-SDS	1.0 ± 0.7	1.4 ± 1.3	0.001	1.0 ± 0.8	1.3 ± 0.8	0.01
Carotid wall cross sectional area (mm^2^)	7.2 ± 1.0	7.4 ± 1.3	0.07	7.4 ± 1.0	7.5 ± 1.3	n.s.
Carotid wall cross sectional area-SDS	0.7 ± 1.1	1.0 ± 1.4	n.s.	0.9 ± 1.2	1.0 ± 1.4	n.s.
Left ventricular mass index (g/m height^2.7^)	34.7 ± 6.1	36.8 ± 6.5	0.008	35.1 ± 4.9	36.8 ± 6.5	0.08
Left ventricular hypertrophy (%)	37/200 (18.5%)	32/91 (35.2%)	0.002; chi-square = 9.602	8/48 (16.7%)	31/89 (34.8%)	*p* = 0.02; chi-square = 5.053

*BMI* body mass index, *DBP* diastolic blood pressure, *SBP* systolic blood pressure, *SDS* standard deviation score

**Fig. 4:**
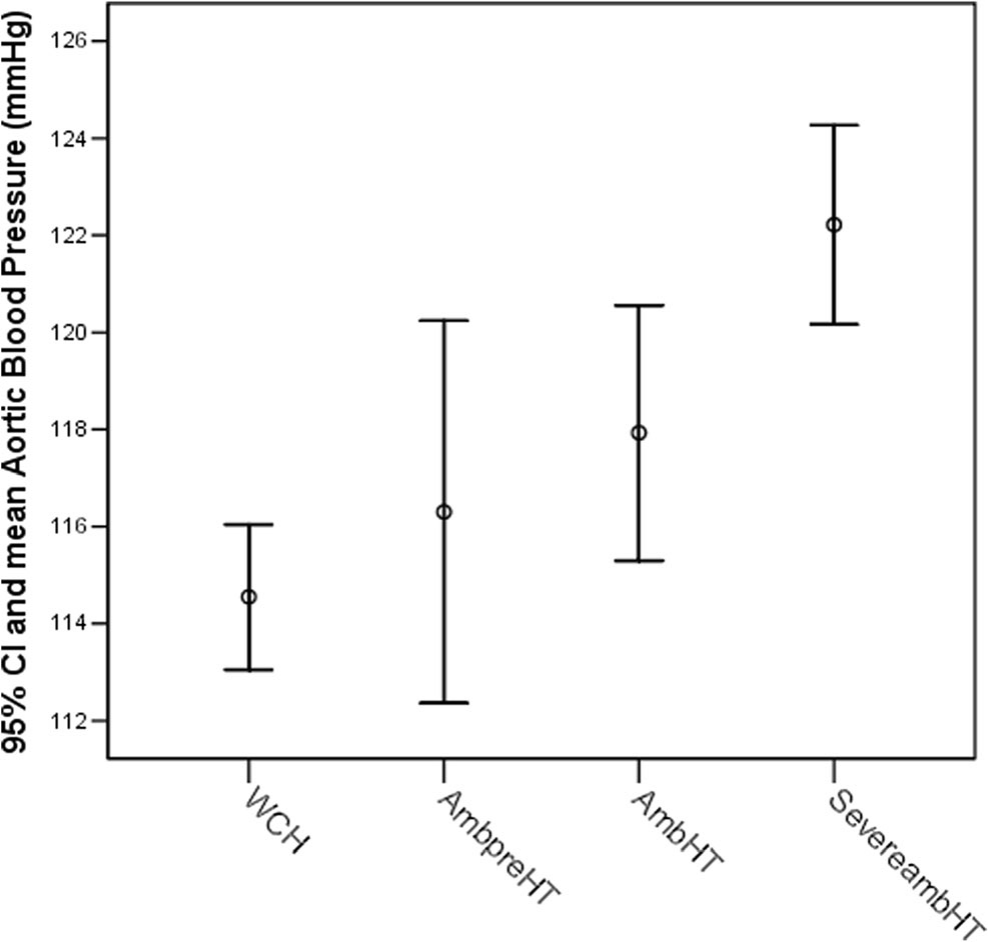
Central systolic blood pressure (cSBP) increase with blood pressure status in ambulatory blood pressure monitoring (ABPM) change. (SevereambHT vs normal ABPM (WCH) and AmbpreHT). (*p* = 0.0001). *WCH* white coat hypertension, *AmbpreHT* ambulatory prehypertension, *AmbHT* ambulatory hypertension, *SevereambHT* severe ambulatory hypertension, *95% CI* 95% confidence interval

cSBP and cPP, cIMT, LVMi, and PWV increased from WCH to SevereambHT with differences found between SevereambHT and WCH patients regarding absolute values of PWV, cSBP, cPP, and cIMT (Table 1, Fig. 4).

There were mild to moderate correlations between indices of cSBP and markers of TOD. cSBP correlated with LVMi (*r* = 0.209; *p* = 0.0001), cIMT (*r* = 0.201; *p* = 0.001), WCSA (*r* = 0.176; *p* = 0.006), PWV (*r* = 0.562; *p* = 0.0001), and PWV-SDS (*r* = 0.378; *p* = 0.0001). cPP correlated with cIMT (*r* = 0.240; *p* = 0.0001), WCSA (*r* = 0.159; *p* = 0.009), and LVMi (*r* = 0.224; *p* = 0.0001). AugPress correlated with LVMi (*r* = 0.224; *p* = 0.0001) and cIMT (*r* = 0.193; *p* = 0.001). AugInd was associated with LVMi (*r* = 0.234; *p* = 0.0001) and cIMT-SDS (*r* = 0.202; *p* = 0.001).

### Predictors of left ventricular hypertrophy

Multivariate regression analysis revealed that the main predictors of left ventricular mass index and LVH were BMI-SDS and WC-SDS (Table 3). However, when anthropometrical variables were excluded from the analysis, AugPress and 24 h SBP were the only predictors of LVMi (Table 4).

**Table 3:** Multivariate regression analysis of predictors of left ventricular mass index

Predictors	*R*	*R* ^2^	Standard error
BMI-SDS	0.395	0.156	5.896
BMI-SDS, WC-SDS	0.480	0.231	5.645

Model with anthropometric variables

*BMI-SDS* body mass index-standard deviation score, *WC-SDS* waist circumference standard deviation score

**Table 4:** Multivariate regression analysis of predictors of left ventricular mass index

Predictors	*R*	*R* ^2^	Standard error
Augmentation pressure	0.275	0.075	6.031
24 h SBP	0.319	0.102	5.955

Model after exclusion of anthropometric variables

*24 h SBP* 24 h systolic blood pressure in ambulatory blood pressure measurement

**Fig. 5:**
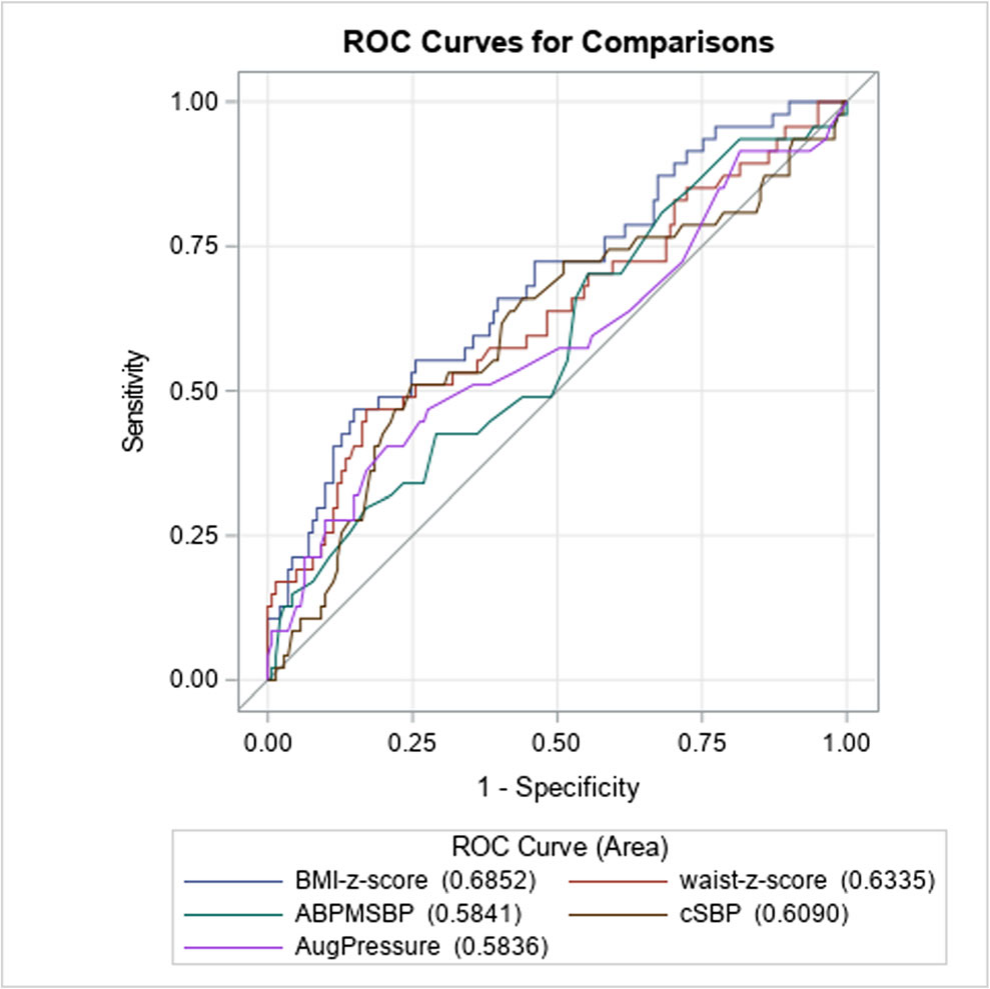
Sensitivity and specificity of cSBP, cPP, AugPress, AugInd, and ABPM SBP as predictors of target organ damage—receiver operator curve analysis. *cSBP* central systolic blood pressure, *cPP* central pulse pressure, *AugPress* augmentation pressure, *AugInd* augmentation index, *ABPM SBP* 24 h systolic blood pressure, *TOD* target organ damage

ROC area for predictors of LVH revealed that cSBP had greater predictive power (0.6090) than 24 h SBP (0.5840). However, markers of obesity (BMI-SDS) and visceral obesity (WC-SDS) had greater area under curve (0.6852 and 0.6335, respectively) (Fig. 5).

## Discussion

The main finding of our study is that one third of children diagnosed according to the current recommendations as PH had normal cSBP and low risk of hypertensive target organ damage. Second, although we found that there is steady increase in cSBP and cPP and of indices of backward wave such as AugPress and AugInd across blood pressure strata from ambulatory normotension to severe ambulatory hypertension, normal cSBP was found even among patients diagnosed with severe ambulatory hypertension. Third, assessment of PWA, including cSBP, cPP, AugPress, and AugInd, had at least the same or higher power as ABPM in predicting presence of hypertensive TOD in children with PH. However, obesity and visceral fatness had even greater predictive value.

There are only few pediatric studies analyzing hemodynamic phenotype and PWA in children with PH. Most of the studies analyzing significance of PWA in assessment of cardiovascular risk in hypertensive patients were performed in adults. In the recently published meta-analysis of studies looking for association between cSBP and hypertensive TOD, it was found that the mean age of a participant was above 40 years. The general conclusion of studies carried out in adults is that cSBP and cPP had better predictive value than brachial SBP and brachial PP in the assessment of hypertensive TOD with the exception of albuminuria^7^. However, it was suggested that the assessment of cSBP and cPP may have greater importance in adolescents and young adults than in the middle age and older people [7, 13, 14]. It is because the middle-sized arteries such as brachial artery are more elastic in children than in adults. It allows for accumulation of backward pulse wave by brachial artery with the rise of brachial SBP but cSBP and cPP do not change. It leads to the increase of SBP in brachial artery and detection of ISH. ISH is a dominant hemodynamic pattern of PH in childhood [28]. In our study, 66% of children with PH confirmed by ABPM had ISH. However, it is not known what is the prevalence of spurious hypertension among children and adolescents diagnosed as PH. Garcia-Espinosa et al. assessed cSBP in 53 children with PH but did not report any case of spurious hypertension [29]. In contrast, Lurbe et al. reported that among hypertensive overweight and obese children 75% of those who had ISH and 50% of those with SDH had normal cSBP and, in fact, had spurious hypertension [30]. When blood pressure status was based on both office and ABPM results, it occurred that 90% of those with SDH had elevated blood pressure both in office, ABPM and cSBP measurements. On the contrary, most of the children with the office ISH had WCH and only 4 out of 19 (21%) with WCH had the elevated cSBP. In contrast, we did not find any case of the elevated cSBP in a group with WCH and in only 2 out of 29 subjects with the ambulatory prehypertension. Also, the prevalence of the elevated cSBP was higher among subjects with ISH (61%) and SDH (75%). The lower prevalence of the elevated cSBP in our group of children with WCH is caused by a threshold value of the elevated cSBP as equal or greater than 95th percentile in our study in contrast to 90th percentile in the study conducted by Lurbe et al. Second, we studied much larger group of the WCH patients and our results may reflect regression to the mean phenomenon. Third, we analyzed a group of patients referred because of the initial diagnosis of PH and much greater number of our patients had confirmed hypertension.

Central systolic blood pressure increased steadily from normal in WCH subjects to elevate in SevereambHT patients. The differences were significant but still 28% of patients with SevereambHT had normal cSBP. Nevertheless, this finding supports rightness of the current pediatric classification of blood pressure status based on ABPM. A diagnosis of hypertension confirmed by ABPM differentiated patients with WCH and sustained hypertension. The same was found also by Lurbe et al. [30]. However, finding that 1/3 of patients with PH confirmed by ABPM had normal cSBP indicates that ABPM alone is insufficient to further discriminate hypertensive patients with a normal and elevated cSBP. Similarly, the pattern of hypertension, i.e., ISH or SDH, did not discriminate between patients with normal cSBP and with elevated cSBP. Although numerically, the prevalence of normal cSBP was greater among patients with ISH in comparison with SDH, it was statistically not significant.

Although cSBP has been linked with cardiovascular events in adults, there are no data on such association from pediatric studies. It is because hypertensive children present the first stages of cardiovascular disease and may present only with subclinical markers of hypertensive TOD such as hypertensive arteriopathy expressed as elevated cIMT and/or WCSA and LVH [6, 31]. cSBP has been found as the main determinant of TOD in adults, both hypertensive and in those with normal peripheral blood pressure [32–35]. There are no studies determining the role of cSBP in TOD in children with PH. Thus, from the practical point of view, it is important to analyze the value of PWA as a marker of TOD in a diagnostic approach in hypertensive children. We found that children who had elevated cSBP had also significantly greater cIMT, LVMi, and PWV values. This finding is similar to the results reported by Totaro et al. who examined 430 normotensive adolescents from the risk group of type 2 diabetes in the mean age of 19.6 years [32]. They found that subjects with higher cSBP had greater cIMT, LVMi, PWV, and lower brachial artery distensibility. However, subjects with higher cSBP had significantly greater BMI (mean BMI 38.7) and 42.9% of them had type 2 diabetes. In contrast, children evaluated in our study were 5 years younger (mean age 15 years), had significantly lower BMI (mean BMI 24.5), and were diagnosed as hypertensive and none of the patients suffered from type 2 diabetes. Multivariate regression analysis revealed that AugPress and 24 h SBP were the main determinants of LVMi and ROC analysis found that cSBP had greater area under curve than ABPM and other parameters of PWA, such as cPP, and AugPress, had the same specificity and sensitivity in predicting LVH as ABPM. Because about 1/3 of children with PH confirmed by ABPM had normal cSBP, these findings suggest that PWA may have complementary role in assessment of cardiovascular risk in hypertensive children. Our findings are also consistent with the idea of spurious hypertension. We found that 39% of adolescents diagnosed with ISH had normal cSBP fulfilling diagnostic criteria of spurious hypertension. Rapid development of portable devices measuring PWV and PWA widens diagnostic opportunities and enables more detailed assessment of hemodynamic phenotype in clinical practice. One must underline that there is potential limitation of interpretation of data obtained from children and adolescents because the transfer function used to calculate cSBP from brachial blood pressure was derived from invasive measurements of cSBP and cPP in adults. On the other hand, results of PWA assessed by oscillometric methods, including Vicorder® were comparable to those obtained with the applanatory tonometry (Sphygmocor) and were validated against invasive measurements of cSBP in adults [16, 17]. Nevertheless, different elastic properties of arterial tree between adolescents and adults may have an effect on the calculation of the transfer function.

According to the current recommendations, pharmacological therapy is suggested in children with PH who had TOD and/or stage 2 hypertension and/or when non-pharmacological therapy is not effective [1, 2]. In view of controversies concerning indications and benefits of a pharmacological therapy in children and young adults with stage 1 PH and without TOD, assessment of PWA may give additional information. However, until further studies confirm our findings and prospective studies show benign nature of ISH in adolescents, it is unclear if the pharmacological treatment should be instituted in adolescents with stage 2 ISH, normal cSBP, and without TOD and other cardiovascular risk factors. Studies in young adults with ISH and normal cSBP showed that during the 10 years of a follow-up, they did not develop sustained hypertension [13]. Also, a large prospective Chicago Heart Study showed that young adult males with ISH had the same risk of cardiovascular events during 31 years long follow-up as those with the blood pressure in high-normal range [36]. However, in this study, cSBP was not assessed. Thus, one can only suppose that some of those subjects with ISH had also normal cSBP. The other issue is the role of sex. Because among hypertensive adolescents, the ratio of boys to girls is 3–4:1, it is important to analyze the cardiovascular risk in relation to sex. The number of girls in our study was too low to reliably analyze the role of sex.

The main weak point of our study is its cross-sectional nature. Thus, it is not possible to assess the risk of development of sustained hypertension and/or TOD in children with spurious hypertension. However, this issue was not studied in children in whom diagnosis of PH was confirmed by ABPM. The strong point of our study is large group of children in whom arterial hypertension was diagnosed and in whom full set of investigations toward TOD were carried out. To the best of our knowledge, it is the largest study to date involving children with arterial hypertension and with full set of data on TOD in whom PWA was assessed.

Although PWA is recommended as the diagnostic approach to hypertensive adults, there are still only few data from pediatric studies. Thus, it is important to evaluate evolution of cSBP, cPP, AugPress, and AugInd during antihypertensive treatment and their relation to a regression of TOD [37]. Second, effects of different antihypertensive drugs and antihypertensive treatment on cSBP should be compared. Third, the potential role of sex on cSBP needs to be determined [38]. Fourth, it is not known what is the evolution of cardiovascular status of children diagnosed as hypertensive but with normal cSBP.

## Conclusions

ABPM alone is not a sufficient tool in a diagnosis of young patients with PH. New methods of cardiovascular risk assessment, such as cSBP, cPP, and AugPress, may help in discriminating patients with sustained hypertension and hypertensive TOD from patients with WCH, ambulatory prehypertension and mild hypertension without TOD. It may be an argument for a wider or even routine use of PWA in the diagnosis and treatment of hypertensive children and adolescents.

## References

[1] Flynn JT, Kaelber DC, Baker-Smith CM, Blowey D, Carroll AE, Daniels SR, de Ferranti SD, Dionne JM, Falkner B, Flinn SK, Gidding SS, Goodwin C, Leu MG, Powers ME, Rea C, Samuels J, Simasek M, Thaker VV, Urbina EM, SUBCOMMITTEE ON SCREENING AND MANAGEMENT OF HIGH BLOOD PRESS IN CHILDREN (2017) Clinical practice guideline for screening and management of high blood press in children and adolescents. Pediatrics 140(3) 10.1542/peds.2017-1904 28827377

[2] Lurbe E, Agabiti-Rosei E, Cruickshank JK, Dominiczak A, Erdine S, Hirth A, Invitti C, Litwin M, Mancia G, Pall D, Rascher W, Redon J, Schaefer F, Seeman T, Sinha M, Stabouli S, Webb NJ, Wühl E, Zanchetti A (2016). 2016 European Society of Hypertension guidelines for the management of high blood pressure in children and adolescents. J Hypertens.

[3] Saba PS, Roman MJ, Pini R, Spitzer M, Ganau A, Devereux RB (1993). Relation of arterial pressure waveform to left ventricular and carotid anatomy in normotensive subjects. J Am Coll Cardiol.

[4] Hashimoto J, Watabe D, Hatanaka R, Hanasawa T, Metoki H, Asayama K, Ohkubo T, Totsune K, Imai Y (2006). Enhanced radial late systolic pressure augmentation in hypertensive patients with left ventricular hypertrophy. Am J Hypertens.

[5] Litwin M, Niemirska A, Sladowska J, Antoniewicz J, Daszkowska J, Wierzbicka A, Wawer ZT, Grenda R (2006). Left ventricular hypertrophy and arterial wall thickening in children with essential hypertension. Pediatr Nephrol.

[6] Litwin M, Niemirska A (2009). Intima-media thickness measurements in children with cardiovascular risk factors. Pediatr Nephrol.

[7] Kollias A, Lagou S, Zeniodi ME, Boubouchairopoulou N, Stergiou GS (2016). Association of central versus brachial blood pressure with target-organ damage: systematic review and meta-analysis. Hypertension.

[8] Roman MJ, Devereux RB, Kizer JR, Lee ET, Galloway JM, Ali T, Umans JG, Howard BV (2007). Central pressure more strongly relates to vascular disease and outcome than does brachial pressure: the Strong Heart Study. Hypertension.

[9] Wang KL, Cheng HM, Chuang SY, Spurgeon HA, Ting CT, Lakatta EG, Yin FC, Chou P, Chen CH (2009). Central or peripheral systolic or pulse pressure: which best relates to target organs and future mortality?. J Hypertens.

[10] Roman MJ, Okin PM, Kizer JR, Lee ET, Howard BV, Devereux RB (2010). Relations of central and brachial blood pressure to left ventricular hypertrophy and geometry: the Strong Heart Study. J Hypertens.

[11] Shimizu M, Kario K (2008). Role of the augmentation index in hypertension. Ther Adv Cardiovasc Dis.

[12] Karamanoglu M, O'Rourke MF, Avolio AP, Kelly RP (1993). An analysis of the relationship between central aortic and peripheral upper limb pressure waves in man. Eur Heart J.

[13] Saladini F, Santonastaso M, Mos L, Benetti E, Zanatta N, Maraglino G, Palatini P, HARVEST Study Group (2011). Isolated systolic hypertension of young-to-middle-age individuals implies a relatively low risk of developing hypertension needing treatment when central blood pressure is low. J Hypertens.

[14] Yano Y, Lloyd-Jones DM (2016). Isolated systolic hypertension in young and middle-aged adults. Curr Hypertens Rep.

[15] Flynn JT, Daniels SR, Hayman LL, Maahs DM, McCrindle BW, Mitsnefes M, Zachariah JP, Urbina EM (2014). American Heart Association Atherosclerosis, Hypertension and Obesity in Youth Committee of the Council on Cardiovascular Disease in the Young Update: ambulatory blood pressure monitoring in children and adolescents: a scientific statement from the American Heart Association. Hypertension.

[16] Pucci G, Cheriyan J, Hubsch A, Hickson SS, Gajendragadkar PR, Watson T, O’Sullivan M, Woodcock-Smith J, Schillaci G, Wilkinson IB, McEniery CM (2012). Evaluation of the Vicorder, a novel cuff-based device for the noninvasive estimation of central blood pressure. J Hypertens.

[17] Hickson SS, Butlin M, Broad J, Avolio AP, Wilkinson IB, McEniery CM (2009). Validity and repeatability of the Vicorder apparatus: a comparison with the SphygmoCor device. Hypertens Res.

[18] Kracht D, Shroff R, Baig S, Doyon A, Jacobi C, Zeller R, Querfeld U, Schaefer F, Wühl E, Schmidt BM, Melk A, 4C Study Consortium (2011). Validating a new oscillometric device for aortic pulse wave velocity measurements in children and adolescents. Am J Hypertens.

[19] Fischer DC, Schreiver C, Heimhalt M, Noerenberg A, Haffner D (2012). Pediatric reference values of carotid-femoral pulse wave velocity determined with an oscillometric device. J Hypertens.

[20] Van Bortel LM, Laurent S, Boutouyrie P, Chowienczyk P, Cruickshank JK, De Backer T, Filipovsky J, Huybrechts S, Mattace-Raso FU, Protogerou AD, Schillaci G, Segers P, Vermeersch S, Weber T, Artery Society; European Society of Hypertension Working Group on Vascular Structure and Function; European Network for Noninvasive Investigation of Large Arteries (2012). Expert consensus document on the measurement of aortic stiffness in daily practice using carotid-femoral pulse wave velocity. J Hypertens.

[21] Herbert A, Cruickshank JK, Laurent S, Boutouyrie P, Reference Values for Arterial Measurements Collaboration (2014). Establishing reference values for central blood pressure and its amplification in a general healthy population and according to cardiovascular risk factors. Eur Heart J.

[22] Marwick TH, Gillebert TC, Aurigemma G, Chirinos J, Derumeaux G, Galderisi M, Gottdiener J, Haluska B, Ofili E, Segers P, Senior R, Tapp RJ, Zamorano JL (2015). Recommendations on the use of echocardiography in adult hypertension: a report from the European Association of Cardiovascular Imaging (EACVI) and the American Society of Echocardiography (ASE). J Am Soc Echocardiogr.

[23] De Simone G, Deveraux RB, Daniels SR, Koren MJ, Meyer RA, Laragh JH (1995). Effect of growth on variability of left ventricular mass: assessment of allometric signals in adults and children and their capacity to predict cardiovascular risk. J Am Coll Cardiol.

[24] Khoury PR, Mitsnefes M, Daniels SR, Kimball TR (2009). Age-specific reference intervals for indexed left ventricular mass in children. J Am Soc Echocardiogr.

[25] Jourdan C, Wühl E, Litwin M, Fahr K, Trelewicz J, Jobs K, Schenk JP, Grenda R, Mehls O, Tröger J, Schaefer F (2005). Normative values of intima-media thickness and distensibility of large arteries in healthy adolescents. J Hypertens.

[26] Doyon A, Kracht D, Bayazit AK, Deveci M, Duzova A, Krmar RT, Litwin M, Niemirska A, Oguz B, Schmidt BM, Sözeri B, Querfeld U, Melk A, Schaefer F, Wühl E, 4C Study Consortium (2013). Carotid artery intima-media thickness and distensibility in children and adolescents: reference values and role of body dimensions. Hypertension.

[27] Elmenhorst J, Hulpke-Wette M, Barta C, Dalla Pozza R, Springer S, Oberhoffer R (2015). Percentiles for central blood pressure and pulse wave velocity in children and adolescents recorded with an oscillometric device. Atherosclerosis.

[28] Sorof JM, Poffenbarger T, Franco K, Bernard L, Portman RJ (2002). Isolated systolic hypertension, obesity, and hyperkinetic hemodynamic states in children. J Pediatr.

[29] García-Espinosa V, Curcio S, Marotta M, Castro JM, Arana M, Peluso G, Chiesa P, Giachetto G, Bia D, Zócalo Y (2016). Changes in central aortic pressure levels, wave components and determinants associated with high peripheral blood pressure states in childhood: analysis of hypertensive phenotype. Pediatr Cardiol.

[30] Lurbe E, Torro MI, Alvarez-Pitti J, Redon P, Redon J (2016). Central blood pressure and pulse wave amplification across the spectrum of peripheral blood pressure in overweight and obese youth. J Hypertens.

[31] Litwin M, Niemirska A, Sladowska-Kozlowska J, Wierzbicka A, Janas R, Wawer ZT, Wisniewski A, Feber J (2010). Regression of target organ damage in children and adolescents with primary hypertension. Pediatr Nephrol.

[32] Totaro S, Khoury PR, Kimball TR, Dolan LM, Urbina EM (2015). Arterial stiffness is increased in young normotensive subjects with high central blood pressure. J Am Soc Hypertens.

[33] Cui R, Li Y, Krisztina G, Yamagishi K, Umesawa M, Imano H, Ohira T, Kiyama M, Okada T, Kitamura A, Hitsumoto S, Tanigawa T, Iso H, Investigators CIRCS (2014). An association between central aortic pressure and subclinical organ damage of the heart among a general Japanese cohort: Circulatory Risk in Communities Study (CIRCS). Atherosclerosis.

[34] Protogerou AD, Argyris AA, Papaioannou TG, Kollias GE, Konstantonis GD, Nasothimiou E, Achimastos A, Blacher J, Safar ME, Sfikakis PP (2014). Left-ventricular hypertrophy is associated better with 24-h aortic pressure than 24-h brachial pressure in hypertensive patients: the SAFAR study. J Hypertens.

[35] DeLoach SS, Daskalakis C, Gidding S, Falkner B (2012). Central blood pressures are associated with left ventricular mass index among African-American adolescents. Am J Hypertens.

[36] Yano Y, Stamler J, Garside DB, Daviglus ML, Franklin SS, Carnethon MR, Liu K, Greenland P, Lloyd-Jones DM (2015). Isolated systolic hypertension in young and middle-aged adults and 31-year risk for cardiovascular mortality: the Chicago Heart Association Detection Project in Industries Study. Am J Cardiol.

[37] Shimizu M, Hoshide S, Ishikawa J, Yano Y, Eguchi K, Kario K (2015). Correlation of central blood pressure to hypertensive target organ damages during antihypertensive treatment: the J-TOP study. Am J Hypertens.

[38] Chester R, Sander G, Fernandez C, Chen W, Berenson G, Giles T (2013). Women have significantly greater difference between central and peripheral arterial pressure compared with men: the Bogalusa heart study. J Am Soc Hypertens.

